# Delusional Parasitosis Without Cutaneous Presentation: “I Have Moths in My Belly”

**DOI:** 10.7759/cureus.63185

**Published:** 2024-06-26

**Authors:** Eduardo D Espiridion, Lily Charron

**Affiliations:** 1 Psychiatry, Drexel University College of Medicine, Philadelphia, USA; 2 Psychiatry, Reading Hospital, West Reading, USA

**Keywords:** somatic symptoms, infestation, psychosis, parasitosis, delusions

## Abstract

Delusional parasitosis is a psychiatric illness characterized by a false belief of a parasite infestation, despite evidence to the contrary. The disorder typically presents as a dermatologic condition since patients often itch and pick at their skin to relieve the perceived infestation. Patients often have numerous cutaneous lesions that never heal due to persistent picking. Another hallmark presentation known as the "matchbox sign" has patients collecting "evidence" of their perceived infestation. This patient believed that he had "moths" infesting his stomach, creating "web-like" structures that spread as far as his nostrils. In this case study, we describe this presentation of the disorder and contextualize our patient in the current literature on delusional parasitosis.

## Introduction

Delusional parasitosis, also known as Ekbom syndrome or delusional infestation, is a rare psychiatric disorder in which a person has a fixed belief that they are infested with parasites, despite concrete evidence to the contrary [1]. In the Diagnostic and Statistical Manual (DSM)-5-TR, a delusional disorder diagnosis includes at least one month of duration of the delusion, a social and occupational functioning that is not markedly impaired, a behavior that is not obviously bizarre or odd, where the symptoms do not meet criteria for schizophrenia, and the condition cannot be explained by other medical or psychiatric illnesses [2]. This is categorized as somatic type and it involves a bodily function and sensation. A typical presentation of delusional parasitosis has cutaneous lesions, where the patient “feels” parasites are under their skin and continues to scratch and pick at the skin for relief. As a result, patients with delusional parasitosis often have several lesions that never heal due to persistent scratching. Another common behavior, known as the “matchbox sign,” is when patients collect “evidence” of the infestation, which may include dirt, skin scrapings, or other particulate matter [3]. Due to the frequency of this cutaneous presentation, most people with delusional parasitosis will present to a general practitioner or dermatologist before seeking psychiatric help.

Delusional parasitosis can be classified as primary or secondary [4]. In the primary disorder, the delusions arise spontaneously. In the secondary disorder, the delusions are due to another neurological, psychiatric, or medical disorder, such as schizophrenia, substance use, depression, or other neuropathies. Psychiatric research estimates the prevalence to be around 40 patients per million people [4]. Though delusional parasitosis is equally common in men and women of most ages, women above the age of 50 are more likely to have the disorder in a 3:1 ratio [3]. Delusional parasitosis is most often comorbid with depression, substance use, and anxiety and one study found about 49% of patients with the disorder to have appearance-related concerns [1,3,5]. Though most cases are idiopathic, about 5-15% of cases of delusional parasitosis are considered shared (also referred to as folie à deux, folie à trois, or folie à famille) with a close relative. Shared delusions tend to present in females and individuals who are submissive and/or socially isolated [6].

Due to the hallmark cutaneous presentation, several differential diagnoses must be considered. Scabies, pet-induced dermatitis, fiberglass dermatitis, substance-induced, pruritus related to systemic disease, schizophrenia, dementia, or other psychiatric disorders should all be considered in the differential diagnoses. The standard treatment for primary delusional parasitosis is antipsychotic medication [4]. Secondary disease can be managed by addressing the primary disorder.

In this article, we present a unique case of delusional parasitosis where the patient does not exhibit cutaneous symptoms. Instead, the patient believes his stomach is infested with moths and “moth-like webs” that spread through his body as far as his nostrils.

## Case presentation

A 59-year-old male was admitted to the medical floor for several medical problems. Among these issues, he complained of persistent abdominal pains which he attributed to moths in his belly. He claimed that these moths fly around a “web-like structure” and that the medical staff needed to give him anti-parasitic medication to get rid of them. He also described the webs coming out of his nose as hair-like threads. He denied any history of psychiatric hospitalization. He also denied any hallucinations, substance abuse, cognitive impairment, vegetative depression symptoms, or suicidal ideation. The patient denied any family history of psychiatric disorders. The patient also reported that he experienced the same symptoms in 1999 and 2008 which were reportedly rectified with anti-parasitic medication, though he could not recall the name of such medication. There were no medical records that corroborated these visits. The patient had never been married or had children. He reported that he needed "placement" as this parasitic infection had caused a significant social functional impairment.

**Figure 1:**
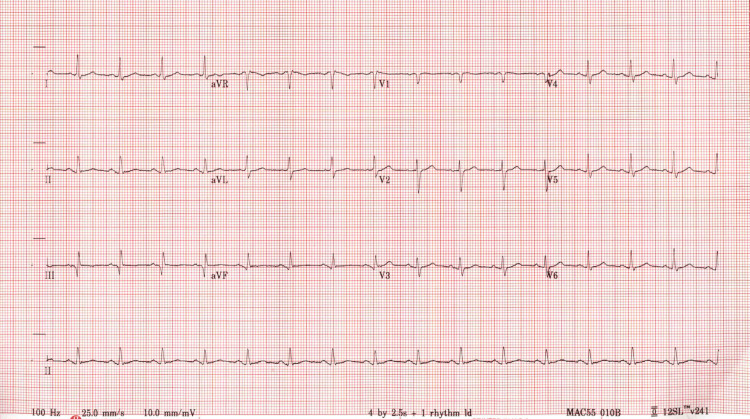
EKG shows elevated Q-T interval and nonspecific ST and T wave abnormality consistent with the patient's history of myocardial infarctions

The patient had a history of several medical problems. He had diabetes mellitus type 2, managed by insulin, and his blood work reflected hyperlipidemia and hyperglycemia (Table 1). The patient also had a history of myocardial infarctions in 2016 and 2023, as evidenced by his EKG (Figure 1).

**Table 1:** Relevant laboratory findings WBC: white blood cells, RBC: red blood cells

Component	Value	Reference range
Glucose	192 mg/dL	74-99 mg/dL
Magnesium	1.5 mg/dL	1.6-2.6 mg/dL
Albumin	3.1 g/dL	3.4-5.0 g/dL
Total Protein	5.3 g/dL	5.7-8.2 g/dL
WBC	5.2 10e^3^/uL	4.8-10.8 10e^3^/uL
RBC	4.91 10e^6^/uL	4.50-6.10 10e^6^/uL
Hemoglobin	14.0 g/dL	14.0-17.5 g/dL
Hematocrit	40.0%	39.0-53.0%
Platelets	149 10e^3^/uL	130-400 10e^3^/uL

**Figure 2:**
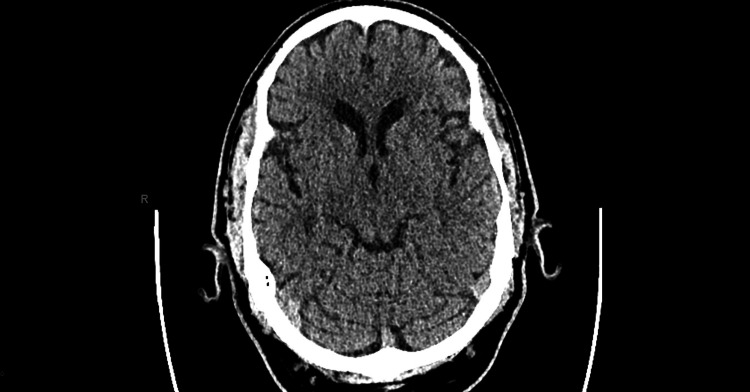
CT scan of the brain; no acute intracranial abnormality

At the present visit, the patient had a thrush infection that was not yet responsive to treatment, slurred speech, and reported numbness on the right side of his face. These symptoms caused concern for a cerebrovascular stroke. However, the patient’s CT scan of the brain (Figure 2) was normal. 

The patient was on several medications: insulin glargine, aspirin, atorvastatin, calcium carbonate, famotidine, fluticasone propionate, lansoprazole, metoclopramide, metoprolol, nitroglycerin, and sodium chloride 0.65%. Once other differential diagnoses were ruled out as possible causes of the patient’s delusions, a psychiatric consult was ordered. At the time of examination, the patient looked appropriate for his stated age. He was fairly groomed as he maintained good eye contact during the interview. There were no rashes or any skin lesions observed. His speech was of normal rate and volume, there was no speech slurring and responses to questions were goal-directed. He denied any suicidal or homicidal ideations, intent, or plans. Cognition and judgment were intact. He had impaired insight and presented with somatic delusions. There were no hallucinations or thought disorganization. The patient did not provide any information that we would suspect a diagnosis of malingering. 

Eventually, a working diagnosis of primary delusional parasitosis was made. There were no agitation or aggressive behaviors observed. The case was discussed with the medical team after they determined that there were no acute medical conditions that could potentially cause the abdominal pains. The patient repeatedly demanded anti-parasitic medications throughout the psychiatric evaluation. The psychiatrist recommended outpatient psychiatric follow-up for medication management. Risperidone 0.5 mg daily with supportive psychotherapy was initiated in the hospital. It was eventually increased to 1 mg. He subsequently noted better sleeping patterns. He tolerated the medication quite well. The patient had a poor social support system and limited collateral information was obtained. The plan was to increase further the risperidone dose slowly in the community. He was referred to the local community mental health center as well as the county case management service.

## Discussion

The lack of any cutaneous manifestation is a presentation of delusional parasitosis that is unique to this patient. It is estimated that more than 80% of cases of delusional parasitosis involve pruritus [6]. The inconsistency between this statistic and the uniqueness of our patient’s presentation in the current literature indicates that delusional parasitosis without cutaneous symptoms may be more common than previously thought.

Though delusions of infection in the stomach are uncommon, there are a few cases with similar presentations. An 82-year-old with no psychiatric history complained of bugs in his stomach and his sputum [7]. This patient reported that the insects originated in the stomach and “crawled” to other organs, including his lungs, causing coughing. This is like the origin and spreading of “moths” in our patient. Unlike our patient, however, this man exhibited several cutaneous symptoms. For one, this patient exhibited frequent itching and vigorous washing of his skin which caused sores. A similar patient reported insects crawling inside her bladder [8]. She awoke one day with tingling from her bladder and believed the cause to be insects, which worsened over a two-year period. Like our patient, she did not report any itching or other cutaneous symptoms. Further, these delusions can be stubborn and long-lasting, such as one case where a female patient reported a parasitic infection for roughly 15 years [9]. She described “little white bugs” crawling in and out of her skin and maintained this to be true despite having a history of psychiatrists, dermatologists, and parasitologists contradicting her delusions.

Hypotheses for the etiology of delusional parasitosis are few and far between. Huber et al. hypothesize that reduced functioning of striatal dopamine transporters may be the key [10]. The study cites several case reports where inhibitors of this transporter elicit the symptoms of delusional parasitosis. Further, several psychiatric disorders involving dopamine, such as schizophrenia, may cause secondary delusions. This hypothesis may also explain the increased prevalence of the disorder with age and the deterioration of dopamine receptor density.

There are several limitations in the research of delusional parasitosis. Delusional parasitosis (whether primary or secondary) can be complicated by comorbid disorders and symptoms. In a report of 50 cases of delusional parasitosis, three of them were diagnosed with leprosy, two with diabetes mellitus, five with depression, two with dementia, and three with trichotillomania [11]. In the cases of diabetes mellitus, like our patient, the onset of the delusions occurred two to three years after diagnosis of diabetes. Further, depression is another common comorbid disorder with delusional parasitosis. In fact, some studies have found antidepressants to alleviate some cases of the disorder [11,12]. Sinha et al. also illustrate a case of delusional parasitosis with several comorbid symptoms [12]. Their patient had a long history of leprosy, vision loss, and iron deficiency anemia, and exhibited depression secondary to the delusions. Interestingly, the cutaneous delusions ceased after treatment of the patient’s anemia. Another example of the diverse presentations of delusional parasitosis is the oral subtype. In one case report of oral delusional parasitosis, the patient presented with several self-inflicted ulcers in the mucosa of the mouth [13]. The patient believed there to be worms under the mucosa. Though a different orifice, this case parallels the presentation of our patient with the moths reaching his nasal mucosa.

The paucity of cases and diversity of presentations means that case studies drive the knowledge base of this disorder. In one systematic review of delusional parasitosis research, most references found were case studies [6]. As the current literature relies on case studies, we hope that the unique presentation of this case will add to the growing picture of this disease.

## Conclusions

Delusional parasitosis is a rare and mostly unknown disorder. The lack of patients, as well as the stigma preventing patients from seeking help, means that research heavily relies on case studies to expand the knowledge base of this disorder. Our patient’s presentation is a unique case where the signature cutaneous symptoms of delusional parasitosis is not present. Instead, the patient’s delusions are confined to moth infestation of his stomach and nares. The patient did not exhibit itching, scabs or lesions, or the matchbox sign which are all typical cutaneous signs. Since most literature on delusional parasitosis lists these cutaneous symptoms as the hallmark of the disease, our case may bring pause to future discussions of this disorder.
